# Substance abuse and personality disorder comorbidity in adolescent outpatients: are girls more severely ill than boys?

**DOI:** 10.1186/s13034-016-0096-5

**Published:** 2016-04-11

**Authors:** Hans Ole Korsgaard, Svenn Torgersen, Tore Wentzel-Larsen, Randi Ulberg

**Affiliations:** Department for Child and Adolescent Mental Health (The Nic Waal Institute), Lovisenberg Diakonale Hospital, Oslo, Norway; Department of Psychology, University of Oslo, Oslo, Norway; Centre for Child and Adolescent Mental Health, Eastern and Southern Norway, Oslo, Norway; Norwegian Centre for Violence and Traumatic Stress Studies, Oslo, Norway; Vestfold Hospital Trust, Tønsberg, Norway; Institute of Clinical Medicine, University of Oslo, Oslo, Norway

**Keywords:** ADHD, Adolescent, Alcohol use disorder, Axis I, Comorbidity, Conduct disorder, Outpatient, Personality disorder, Substance use disorder

## Abstract

**Background:**

Substance use disorders (SUDs) constitute a major health problem and are associated with an extensive psychiatric comorbidity. Personality disorders (PDs) and SUDs commonly co-occur. Comorbid PD is characterized by more severe addiction problems and by an unfavorable clinical outcome. The present study investigated the prevalence of SUDs, PDs and common Axis I disorders in a sample of adolescent outpatients. We also investigated the association between PDs and SUDs, and how this association was influenced by adjustment for other Axis I disorders, age and gender.

**Methods:**

The sample consisted of 153 adolescents, aged 14–17 years, who were referred to a non-specialized mental health outpatient clinic with a defined catchment area. SUDs and other Axis I conditions were assessed using the mini international neuropsychiatric interview. PDs were assessed using the structured interview for DSM-IV personality.

**Results:**

18.3 % of the adolescents screened positive for a SUD, with no significant gender difference. There was a highly significant association between number of PD symptoms and having one or more SUDs; this relationship was practically unchanged by adjustment for gender, age and presence of Axis I disorders. For boys, no significant associations between SUDs and specific PDs, conduct disorder (CD) or attention deficit hyperactivity disorder (ADHD) were found. For girls, there were significant associations between SUD and BPD, negativistic PD, more than one PD, CD and ADHD.

**Conclusions:**

We found no significant gender difference in the prevalence of SUD in a sample of adolescents referred to a general mental health outpatient clinic. The association between number of PD symptoms and having one or more SUDs was practically unchanged by adjustment for gender, age and presence of one or more Axis I disorders, which suggested that having an increased number of PD symptoms in itself may constitute a risk factor for developing SUDs in adolescence. The association in girls between SUDs and PDs, CD and ADHD raises the question if adolescent girls suffering from these conditions may be especially at risk for developing SUDs. In clinical settings, they should therefore be monitored with particular diligence with regard to their use of psychoactive substances.

*Trial registration* The regional committee for medical research ethics for eastern Norway approved the study protocol in October 2004 (REK: 11395). Address correspondence and reprint requests to: Hans Ole Korsgaard, The Nic Waal Institute, Lovisenberg Diakonale Hospital, P.O. Box 2970 Nydalen, N-0440 Oslo, Norway; E-mail hansole.korsgaard@tele5.no

## Background

Personality disorders (PDs) are defined as enduring and maladaptive patterns of experiencing, coping, and relating to others. In DSM-IV, as well as DSM-5, PD categories may be applied to adolescents when the individual’s particular maladaptive personality traits appear to be pervasive, persistent, and unlikely to be limited to a particular developmental state or an episode of an Axis I disorder. With the exception of antisocial PD (ASPD), any PD can be diagnosed in a person under 18 years of age, as long as the diagnostic features have been present for at least 1 year [1, 2].

PDs are common conditions, with prevalences of about 13 % in the general adult population, up to 40 % in adult outpatient samples, and up to 71 % in inpatient samples when diagnosed with comprehensive semi-structured interviews [3]. In adolescents, prevalences range from 6 to 17 % in community samples, and in clinical samples from 41 to 64 % [4]. Pathological personality traits emerge at an early age and are related to health-risk behaviors in adolescence as well as young adulthood [5–7], but PD diagnoses may be less stable than previously assumed [8]. Maladaptive personality trait constellations, however, seem to be more stable in their structure than PD diagnoses. They may change in severity or expression over time; still they often lead to persistent functional impairment and reduced quality of life, even if the diagnostic threshold for a specific PD is no longer reached [9, 10].

Borderline PD is the single most studied PD, and is generally considered as the prototypical cluster B disorder. BPD may be more prevalent than previously recognized, with a lifetime prevalence of up to 2.7 % in the general adult population [11]. A large population study found BPD equally prevalent among men and women, and frequently associated with considerable mental and physical disability, especially among women [12]. There is an increasing awareness of developmental antecedents and adolescent presentation of BPD [13–15], with several studies pointing out prognostic advantages of early identification and timely treatment of PDs [16, 17]. It has recently been shown that the diagnosis of BPD is as reliable and valid in adolescents as it is in adults, and that adolescents with BPD can benefit from early intervention [18].

Substance use disorders (SUDs) constitute a major health problem, with estimated prevalence rates of 3.4 % for alcohol dependence and 0.3–1.8 % for cannabis dependence in the general European population [19]. It has generally been assumed that boys use more drugs and alcohol than girls. However, recent findings seem to contradict this long-held assumption; Johnson and colleagues found that male–female differences in adolescent marijuana use have decreased since 1999 [20], and another study reports that the differences in drinking patterns of adolescent boys and girls narrowed between 2002 and 2012 [21]. Drug abuse is associated with an extensive psychiatric comorbidity and carries an increased risk of premature death, especially in male users of opiates or barbiturates [22]. Estimated lifetime prevalences of SUDs in adolescents and young adults range from 4.6 [23] to 17.7 % [24]. In adolescents, SUDs are of considerable importance in the etiology and prognosis of psychiatric disorders such as mood disorders, conduct disorder (CD), attention-deficit hyperactivity disorder (ADHD), and anxiety disorders [25]. In adults, generalized anxiety disorder (GAD) and SUDs are highly comorbid, and GAD–SUD comorbidity is associated with a host of poor psychosocial outcomes, including higher rates of hospitalization, disability, functional impairment, and inferior GAD and SUD treatment outcomes [26].

Adolescents with SUDs tend to have higher rates of comorbid psychiatric disorders and are more likely to report a history of trauma and physical and/or sexual abuse than adolescents without a SUD [27, 28]. In addition, psychiatric disorders in adolescents often predate the SUD. Once the SUD develops, the psychiatric disorder may be further exacerbated [29] and associated with substantial functional impairment [30]. In older adolescence and emerging adulthood, young drug users with comorbid affective disorders have greater mental health and substance use morbidity than those with substance use problems alone [31]. A study of adolescent SUD inpatients found that 40.5 % of the participants fulfilled criteria for at least one comorbid present Axis I disorder, with high prevalences of mood, anxiety, and somatoform disorders. The 37 female participants showed a significantly higher risk for lifetime comorbid disorders; the gender difference was especially pronounced for anxiety and somatoform disorders [32].

ADHD has been shown to be a significant risk factor for developing SUDs [33]. It is frequently present in SUD populations, with prevalence estimates varying between 14 and 23 %. In general, patients with this type of comorbidity represent a more severe subgroup of SUD patients with more additional comorbidity and a more disadvantageous prognosis than SUD patients without ADHD [34]. It has been suggested that girls with ADHD might be at slightly higher risk than boys for substance abuse [35]. CD is a risk factor of similar magnitude as ADHD, and of equal importance in both genders [35].

PDs and SUDs commonly co-occur, with many studies finding a particularly frequent association between SUDs and BPD or ASPD [25, 36–38]. Comorbid PD seems to be more prevalent in drug use disorder (DUD) than in alcohol use disorder (AUD) [37]. Comorbid PD is characterized by more severe addiction problems and by an unfavorable clinical outcome [39]. Prevalence rates of PDs in patients with SUD range from 24 to 90 %, depending on the sample characteristics and setting [11, 40–42]. A Norwegian study of first-admission SUD patients aged 16 years and older, found that 46 % of the patients had at least one PD. In this sample, cluster C disorders were as prevalent as cluster B disorders; SUD patients with PDs were younger at the onset of their first SUD and at admission; they used more illicit drugs; had more anxiety disorders; had more severe depressive symptoms; were more distressed and more impaired in their social functioning [37]. Comorbid SUD can be diagnosed in approximately every second patient suffering from a PD [36].

Some studies have reported gender differences in adolescents and young adults; Foster and colleagues found AUD to be a more severe disorder in women than in men. Despite lower mean levels of overall risk exposure, women were characterized by higher levels of adolescent risk factors and a greater magnitude of AUD consequences. Furthermore, internalizing symptoms appeared to be a gender-specific risk factor for AUD in women [43]. Roberts and colleagues found a tendency in females with SUDs to have higher rates of comorbid disorders, as did older youths [30]. Thus, the question of possible gender differences in SUD prevalence, comorbidity and prognosis has not yet been fully answered.

## Aims

The objective of the present study, performed on a clinical sample of consecutively referred adolescent outpatients, was to

1. Investigate the prevalences of alcohol and substance abuse and common Axis I disorders, including possible gender differences.
2. Investigate the association between PDs and alcohol and other substance abuse. We also wanted to assess the influence of adjusting for other Axis I disorders, age and gender on this association.

## Methods

### Participants

The present study used a sample of adolescents aged 14–17 years who were referred to a mental health outpatient clinic for children and adolescents in Oslo (The Nic Waal Institute, Lovisenberg Diakonale Hospital). The catchment area of the clinic comprises 25.000 children and adolescents from 0 to 17 years of age, and consists of four city districts with a population of mixed socioeconomic status, representing all social classes including immigrant workers and well-educated middle and upper class families. Study inclusion took place from February 2005 to April 2007. All referred patients in the study’s age group were asked to participate. Exclusion criteria were the need for immediate hospitalization or other urgent therapeutic measures, clinically assessed mental retardation, lack of fluency in the Norwegian language, and absence of the evaluator at the time of referral [44].

### Measures

As in other comparable studies on the prevalence of Axis I and Axis II disorders in adolescents, well validated adult diagnostic tools have been used [45–48].

### Axis I disorders

Axis I disorders, including SUDs, were assessed using a Norwegian translation of the mini international neuropsychiatric interview version 5.0.0 (MINI) [49, 50]. The MINI has not been validated for adolescents, but has previously been used in studies on adolescents [51] and was chosen for its excellent feasibility [50].

In the assessment of ADHD a primary screening was first performed, using the six-item adult ADHD Self-Report Scale Screener version 1.1 (ASRS Screener) in a Norwegian version [52]. The ASRS Screener is reliable and valid in adult clinical settings, with excellent specificity [53]. It has repeatedly been shown to be in strong concordance with clinician diagnoses [54]. The ASRS Screener has not been validated for use in adolescents, but the full 18-item ASRS symptom checklist, from which it is derived, has been found to be reliable and valid in adolescents [55].

If the primary screening with the ASRS Screener was positive, the Mini International Neuropsychiatric Interview-PLUS (MINI-PLUS) section W (ADHD in children/adolescents) was used as a diagnostic test instrument [50] for a final diagnosis of ADHD.

### Personality disorders

The Structured Interview for DSM-IV (SIDP-IV) [56] in a Norwegian version was used to assess PDs. The SIDP-IV is a comprehensive semi-structured diagnostic interview for DSM-IV PD (Axis II) diagnoses, which has been used in numerous studies in different countries, including Norway [57–59]. The SIDP-IV has been extensively used in research on PDs in adolescence [51, 60, 61]. The SIDP-IV covers 14 DSM-IV Axis II diagnoses as well as CD as a separate axis I disorder. The Axis II diagnoses comprise the ten standard DSM-IV PDs (paranoid, schizoid, schizotypal, borderline, histrionic, narcissistic, antisocial, obsessive–compulsive, dependent, and avoidant PD), the three provisional DSM-IV PDs (self-defeating, depressive, and negativistic PD), and mixed PD.

All questions address the typical or habitual behavior of the subjects during the last 5 years. Each diagnostic criterion is rated on a four point scale: “0” = criterion not present; “1” = subthreshold level of the trait present; “2” = criterion being present for most of the last 5 years; and “3” = criterion strongly present. Scores “2” and “3” indicate the presence of a criterion according to DSM-IV [56]. In the following text, we will be using the term “PD symptoms” when a diagnostic criterion meets a score of 1, 2 or 3. “PD” is used when a sufficient number of diagnostic criteria for a specific DSM-IV diagnosis are fulfilled, as measured with the SIDP-IV.

In accordance with diagnostic practice applied in other studies on PDs in adolescence, the DSM-IV age criterion for ASPD was waived [45]. Due to the participants’ age, we also waived the 5 year symptom duration criterion. Instead we used 2 years symptom duration as criterion. This is in accordance with the criterion used in previous studies assessing adolescent personality pathology [4, 45].

### Procedures and assessment

All patients were assessed immediately upon referral by the first author, who was a male specialist in psychiatry and child and adolescent psychiatry, with 21 years of clinical experience. He was trained in evaluation with SIDP-IV by the second author, who was an experienced rater, who had previously evaluated patients and reported from comparable studies in adults [59, 62]. Twenty ratings were discussed and found to be in accordance with the rating of the experienced evaluator. Axis I conditions were also assessed by the first author, who had been trained by the translator of the Norwegian version of the MINI.

After completion of the initial assessment, the patients were assigned to further clinical evaluation and treatment by clinicians other than the first author in the outpatient clinic.

### Statistical analysis

Descriptive statistics were calculated for the relevant mental health status variables and expressed in mean [with standard deviation (SD) in parentheses] and frequency (percentages in parentheses) as appropriate. Prevalences of PDs, SUDs and other Axis I conditions with 95 % Blaker confidence intervals [63] were estimated for the total sample and for each gender separately, with testing for gender differences by exact Chi square tests. SUD was classified as none, one [either AUD or cannabis use disorder (CUD)] and two (both AUD and CUD). The association of SUD with number of PD symptoms, unadjusted and adjusted for gender, age and presence of Axis I disorders was investigated by proportional odds ordinal logistic regression. Differences in unadjusted and adjusted odds ratios were, if necessary, investigated by a bootstrap BC_a_ 95 % confidence intervals based on 10,000 bootstrap replicates [64], with a difference considered as significant if 0 was outside the interval. Data were analysed using the IBM SPSS version 20.0 software, with Blaker confidence intervals and bootstrapping using the R (The R Foundation for Statistical Computing, Vienna, Austria) packages BlakerCI and boot.

### Ethical statement

The study was approved by the regional committee for medical research ethics for eastern Norway (REK: 11395) and by The Norwegian Data Inspectorate. Informed written consent was obtained from all patients, and for patients younger than 16 years consent was additionally obtained from their parents.

## Results

In the study inclusion period a total of 264 adolescents (59.4 % female) were referred to The Nic Waal Institute. Sixty-three patients did not meet the inclusion criteria; they were excluded due to inadequate fluency in the Norwegian language (N = 6, 9.5 %), mental retardation (N = 15, 23.8 %), need of immediate hospitalization (N = 19, 30.2 %), and absence of the evaluator at the time of referral (N = 23, 36.5 %). This left 201 adolescents eligible for inclusion in the study. The attrition was 48 (23.9 %); lack of consent from parents (N = 5, 10.4 %), referral retracted prior to interview (N = 6, 12.5 %), lack of consent from the adolescent (N = 7, 14.6 %), did not show up for appointment (N = 11, 22.9 %), and consent retracted during interview (N = 19, 39.6 %) [44].

A total of 153 adolescents (61.4 % girls, mean age 16.0 years; SD = 1.1, range 14.1–18.0 years) were finally included in the study. There were no missing data in any items within the ASRS Screener, MINI, MINI-PLUS section W, or SIDP-IV.

Of the adolescents, 18.3 % (N = 28, 95 % CI 12.6–25.3 %) were diagnosed with a SUD using the MINI, with no significant gender difference in prevalence (Table 1). Apart from alcohol, cannabis was the only drug in the sample that qualified for either an abuse or a dependency diagnosis. When analysed separately for alcohol and cannabis problems in each gender, boys had slightly more alcohol-related problems, whereas girls had slightly more cannabis-related problems; the differences were not significant (alcohol; χ^2^ = 0.027, p = 1.000, cannabis χ^2^ = 0.055, p = 1.000). The female/male ratio of SUDs was 1.16 (95 % CI = 0.49–2.72, p = 0.73).

**Table 1:** Prevalence of SUD, other Axis I disorders and personality disorders (N = 153)

	Boys (N = 59) N (%) (CI^a^)	Girls (N = 94) N (%) (CI^a^)	Total (N = 153) N (%) (CI^a^)	p value^b^
Without SUD	49 (83.1 %) (71.5–91.3 %)	76 (80.9 %) (71.5–88.1 %)	125 (81.7 %) (74.6–87.3 %)	–
With SUD	10 (16.9 %) (8.7–28.5 %)	18 (19.1 %) (11.9–28.5 %)	28 (18.3 %) (12.6–25.3 %)	0.831
With AUD	7 (11.9 %) (5.38–22.5 %)	10 (10.6 %) (5.46–18.3 %)	17 (11.1 %) (6.73–17.1 %)	1.000
With CUD	7 (11.9 %) (5.38–22.5 %)	12 (12.8 %) (7.08–21.0 %)	19 (12.4 %) (7.93–18.5 %)	0.540
Anxiety	13 (22.0 %) (13.0–34.5 %)	38 (40.4 %) (30.7–50.7 %)	51 (33.3 %) (26.0–41.1 %)	*0.022*
Mood	13 (22.0 %) (13.0–34.5 %)	37 (39.4 %) (29.6–49.6)	50 (32.7 %) (25.3–40.5 %)	*0.033*
Psychosis	0 (0.0 %) (0.0–6.0 %)	2 (2.1 %) (0.4–7.1 %)	2 (1.3 %) (0.2–4.6 %)	0.523
OCD	4 (6.8 %) (2.3–16.4 %)	10 (10.6 %) (5.5–18.3 %)	14 (9.2 %) (5.3–14.8 %)	0.568
CD	12 (20.3 %) (11.3–32.8 %)	15 (16.0 %) (9.5–24.8 %)	27 (17.6 %) (12.2–24.4 %)	0.519
ADHD	9 (15.3 %) (7.9–26.8 %)	12 (12.8 %) (7.1–21.0 %)	21 (13.7 %) (8.9–20.1 %)	0.810
PD diagnosis	8 (13.6 %) (1.3–7.3 %)	25 (26.6 %) (6.0–24.4 %)	33 (21.6 %) (15.5–28.6 %)	0.070
No diagnosis^c^	23 (39.0 %) (26.8–52.2 %)	28 (29.8 %) (21.0–39.8 %)	51 (33.3 %) (26.0–41.1 %)	0.168

*SUD* substance use disorders: alcohol and/or drug abuse or dependence. SUD is equivalent to AUD and/or CUD, since no other substances were used in our data; *AUD* alcohol use disorders: alcohol abuse or dependence; *CUD* Cannabis use disorders: Cannabis abuse or dependence; *Anxiety* anxiety disorders: simple phobias, generalized anxiety disorder, panic disorder, agoraphobia, social phobia and post-traumatic stress disorder; *Mood* mood disorders: dysthymia and major depressive episode; *OCD* obsessive–compulsive disorder; *CD* conduct disorder; *ADHD* attention deficit hyperactivity disorder

^a^Blaker 95 % confidence intervals

^b^ *p* value from exact Chi square test

^c^ *No diagnosis* no Axis I or personality disorder diagnosis

Two thirds (63.4 %, N = 97) of the adolescents met the criteria for at least one Axis I disorder (68.1 %, N = 64 girls; 56.0 %, N = 33 boys). Anxiety disorders; simple phobias, GAD, panic disorder, agoraphobia, social phobia and post-traumatic stress disorder (33.3 %, N = 51, 95 % CI 26.0–41.1 %) and mood disorders; dysthymia and major depressive episode (32.7 %, N = 50, 95 % CI 25.3–40.5 %) were most frequent, followed by SUD (18.3 %, N = 28, 95 % CI 12.6–25.3 %), CD (17.6 %, N = 27, 95 % CI 12.2–24.4 %), obsessive–compulsive disorder (9,2 %, N = 14, 95 % CI 5.3–14.8 %) and psychotic disorders (1.3 %, N = 2, 95 % CI 0.2–4.6 %). There were significant gender differences in anxiety (p = 0.022) and mood (p = 0.033) disorders (Table 1).

Of the adolescents, 21.6 % (N = 33) had at least one PD, 7.2 % (N = 11) had more than one PD, and 4.6 % (N = 7) had both ADHD and a PD. The prevalence of PDs was generally higher in the referred girls. Girls showed significant associations between SUD and BPD (p = 0.024), negativistic PD (p = 0.035), more than one PD (p = 0.020) as well as between SUD and CD (p = 0.001) and ADHD (p < 0.001) (Table 2).

**Table 2:** Prevalence of specific personality disorders, conduct disorder, and ADHD in adolescents with SUD (N = 153)

Personality disorder (PD)	Boys with SUD (N = 10)	Boys without SUD (N = 49)	χ^2^	p value^a^	Girls with SUD (N = 18)	Girls without SUD (N = 76)	χ^2^	p value^a^
Paranoid	0	0	–	–	0	0	–	–
Schizoid	0	1	0.183	1.000	0	0	–	–
Schizotypal	0	0	–	–	0	0	–	–
Antisocial	1	1	1.606	0.313	1	2	4.519	0.093
Borderline	1	0	4.984	0.169	4	3	7.052	*0.024*
Histrionic	0	0	–	–	2	3	1.483	0.243
Narcissistic	0	0	–	–	0	1	0.239	1.000
Avoidant	0	3	0.645	0.638	1	5	0.026	1.000
Dependent	0	0	–	–	0	1	0.239	1.000
Obsessive–compulsive	0	0	–	–	0	6	1.518	0.350
Self-defeating	0	0	–	–	0	0	–	–
Depressive	0	2	0.422	1.000	2	6	0.193	1.000
Negativistic	0	0	–	–	2	0	8.628	*0.035*
At least one PD	1	7	0.130	1.000	7	18	1.723	0.237
More than one PD	1	0	4.984	0.169	5	5	6.880	*0.020*
Conduct disorder	4	8	2.873	0.189	8	7	13.472	*0.001*
ADHD	2	7	0.210	1.000	8	4	20.062	<*0.001*

*SUD* substance use disorders: alcohol and/or drug abuse or dependence

^a^p values from exact Chi square tests

**Fig. 1:**
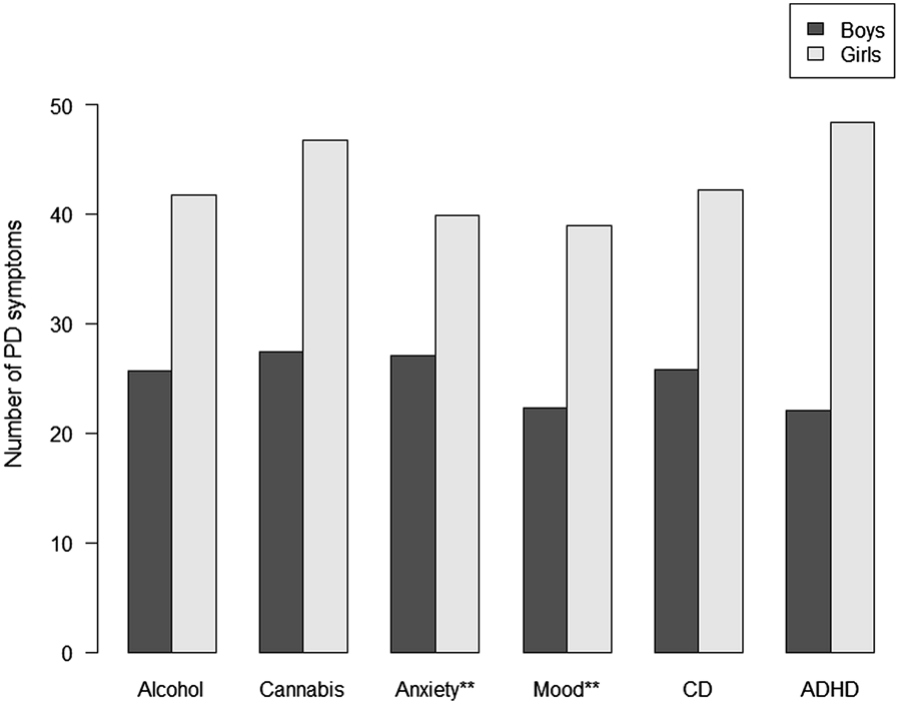
PD symptoms in adolescents with SUD and other Axis I disorders. *PD Symptoms* any PD criteria meeting a score of 1,2 or 3 when measured with the SIDP-IV; *SUD* substance use disorders; alcohol and/or drug abuse or dependence. *Alcohol* alcohol abuse or dependence; *Cannabis* Cannabis abuse or dependence; *Anxiety* anxiety disorders, simple phobias, generalized anxiety disorder, panic disorder, agoraphobia, social phobia and post-traumatic stress disorder; *Mood* mood disorders, dysthymia and major depressive episode; *CD* conduct disorder; *ADHD* attention deficit hyperactivity disorder. ** p < 0.05

Figure 1 illustrates the association between PD symptoms and SUD and other frequent Axis I disorders. As can be seen, girls had more symptoms than boys in all reported Axis I conditions; the difference was significant for anxiety disorders (p = 0.022) and mood disorders (p = 0.033). Substance disorders (p = 0.831) and CD (p = 0.585) did not yield significant gender differences.

There was a significant positive association between the number of PD symptoms and SUD (OR per five points difference in the number of PD symptoms 1.16, 95 % CI 1.06–1.26, p = 0.001). The association was still significant after adjustment for gender, age and presence of one or more Axis I disorders (OR 1.15, 95 % CI 1.04–1.27, p = 0.005). There were no significant deviations from the proportional odds assumption in these analyses (p ≥ 0.466). No bootstrap procedure for comparing the unadjusted and adjusted ORs was performed due to the almost total overlap between the confidence intervals.

## Discussion

The present study investigated the prevalence of SUDs and common Axis I disorders in an unselected sample of adolescents. The participants were all referred to a non-specialized mental health outpatient clinic with a defined catchment area. We also investigated the association between PD symptoms and SUDs, as well as how this relationship was influenced by adjustment for other Axis I disorders, age and gender.

Our finding of 18.3 % of the adolescents having AUD or CUD seems not to be incongruent with previous findings, considering that studies of non-referred adolescents have found SUD prevalence rates of 4.6 % [23] to 17.7 % [24], and the prevalence rate in adolescent and young adult inpatients has been reported to be up to 54 % for DUD and 87 % for AUD when first admitted to hospital treatment [37]. As was to be expected, the participants in the present study had a higher prevalence of SUDs than has been found in studies of community samples and primary care patients but a lower prevalence than that seen in participants in studies of more severely ill patients.

An earlier study of adolescents has reported significantly higher risk for lifetime comorbid disorders in women, with an especially pronounced gender difference for anxiety and somatoform disorders [32]. In the present study, however, significant gender differences in anxiety and mood disorders were found only in the adolescents that did not have SUDs.

It has been suggested that girls with ADHD might be at slightly higher risk than boys for substance abuse [35]. In accordance with this, the present study found significantly more ADHD as well as CD in girls than in boys with SUDs.

Recent findings have contradicted the assumption that boys generally use more drugs and alcohol than girls [20, 21]. Our findings of non-significant differences between genders in SUD prevalence are in accordance with this trend. Other recent studies have reported AUD to be a more severe disorder in adolescents and young adults, with higher levels of adolescent risk factors and a greater magnitude of AUD consequences in women [43], as well as a tendency in females with SUDs to have higher rates of comorbid disorders [30]. The cross-sectional nature of the present study makes it impossible to infer causal relationships, but our findings do support the assumption of a more extensive psychiatric comorbidity in female adolescent SUD patients.

The main finding of the present study is a highly significant association between number of PD symptoms and the presence of one or more SUDs (p = 0.001), with almost totally overlapping confidence intervals after adjustment for gender, age and presence of one or more Axis I disorders (p = 0.005). This finding implies that having an increased number of PD symptoms in itself is a unique risk factor for the later development of a SUD.

## Strengths and limitations

The study was performed at a single general service mental health outpatient clinic, receiving adolescents from a geographically defined urban area with a varied socioeconomic and ethnic population. Still, the results from the present study may not be generalizable to other outpatient populations. The participants were included in a limited time span, and we cannot exclude the possibility of prevalence fluctuations over time.

The attrition (23.9 %, N = 48) and the relatively small sample size also constitute limitations. In particular, the sample size limits the degrees of freedom available, so that analysis of single PDs in some cases may be statistically underpowered. Therefore, we have mainly focused on the total number of PD symptoms rather than on specific PDs. This might constitute a limitation. However, in light of current epidemiological knowledge about PDs, the differentiation between having or not having a PD is clearly more relevant than the differentiation between specific PDs. It should also be pointed out that specific PD diagnoses merely reflect the presence of an arbitrarily stipulated number of PD symptoms; there is no indication whatsoever of the existence of categorical breaking points at a given number of PD symptoms. On the contrary, recent literature supports the notion of PDs as dimensional entities with arbitrarily defined diagnostic cut-off points deciding whether or not a patient is above the diagnostic threshold for a specific disorder [4, 65].

The gender distribution of our sample was close to identical to the gender distribution of all referred adolescents in the study inclusion period, and reflects the fact that in adolescence, as opposed to middle and late childhood, more girls than boys are referred to Norwegian mental health outpatient clinics.

Each patient was diagnosed individually with well-documented and semi-structured test instruments by a single, experienced clinician and rater. Due to the fact that just one person performed all assessment work, there was no missing data. The evaluator was trained in rating with SIDP-IV and MINI by experienced evaluators and researchers on PD and Axis I diagnoses. Notwithstanding, the use of a single evaluator constitutes a possible limitation; it may have strengthened the internal validity, but might have been a threat to the external validity of the diagnoses.

## Conclusions

The present study comprised 153 adolescents referred to a non-specialized mental health outpatient clinic. Of these adolescents, 18.3 % screened positive for AUD or CUD, with no significant gender difference in prevalence. The female/male ratio of SUDs was 1.13 (95 % CI = 1.10–1.17). More than two-thirds of the adolescents met the criteria for at least one Axis I disorder, with significant gender differences in anxiety (p = 0.022) and mood (p = 0.033) disorders; 21.6 % of the patients had at least one PD and 7.2 % had more than one PD. The prevalence of PDs was generally higher in the referred girls. For boys, no significant associations between SUDs and specific PDs or Axis I disorders could be ascertained. For girls, there were significant associations between SUD and BPD, negativistic PD, more than one PD, CD and ADHD.

There was a highly significant association between number of PD symptoms and the presence of one or more SUDs. This association was practically unchanged when adjusted for gender, age and having one or more Axis I disorders, suggesting that having an increased number of PD symptoms in itself may constitute a unique risk factor for developing SUDs in adolescence. These findings are in accordance with earlier reports of increased co-occurrence of PDs and SUDs in adolescence [36–38].

However, the girls in the study were overall more severely ill than the boys; girls with SUDs differed even more so, with significant associations between SUDs and BPD (p = 0.024), negativistic PD (p = 0.035), more than one PD (p = 0.020), as well as between SUDs and CD (p = 0.001) and ADHD (p < 0.001). This indicates that adolescent girls suffering from these disorders may be especially at risk for developing SUDs. In clinical practice, it might therefore be suggested that girls presenting with BPD, negativistic PD, more than one PD, ADHD, or CD should be monitored with particular diligence with regard to their use of psychoactive substances.

## Abbreviations

ADHD: attention deficit hyperactivity disorder
ASPD: antisocial personality disorder
AUD: alcohol use disorder
BPD: borderline personality disorder
CD: conduct disorder
CUD: Cannabis use disorder
DUD: drug use disorder
GAD: generalized anxiety disorder
ODD: oppositional defiant disorder
PD: personality disorder
SUD: substance use disorder
